# A novel splice site mutation in the *COL4A5* gene in a Chinese female patient with rare ocular abnormalities

**Published:** 2012-08-08

**Authors:** Chan Zhao, Fang Wang, Yanqin Zhang, Yubing Wen, Ying Su, Chengfen Zhang, Ruifang Sui, Fei Xu, Jie Ding, Fangtian Dong

**Affiliations:** 1 Department of Ophthalmology, Peking Union Medical College Hospital, Chinese Academy of Medical Sciences & Peking Union Medical College, Beijing, P.R. China; 2 Department of Pediatrics, Peking University First Hospital, Beijing, P.R. China; 3 Department of Nephrology, Peking Union Medical College Hospital, Chinese Academy of Medical Sciences & Peking Union Medical College, Beijing, P.R. China

## Abstract

**Purpose:**

To describe an unusual ocular phenotype in a Chinese female patient with X-linked Alport syndrome (XLAS), and to characterize the type IV collagen alpha 5 (*COL4A5*) gene mutation in the patient and her son.

**Methods:**

Detailed ophthalmologic examinations and optical coherence tomography were performed in the patient and her family members. For gene analysis of *COL4A5*, the entire coding region of *COL4A5* mRNA from cultured skin fibroblast was analyzed by using reverse-transcription-polymerase chain reaction (RT–PCR) and direct sequencing, and genomic DNA was analyzed by using PCR and direct sequencing.

**Results:**

The patient presented with progressive myopia at age 14 and bilateral giant macular holes (about 2 disc diameter) at age 28. At age 33 when presented to our hospital, slit lamp examination of the anterior segment showed bilateral anterior and posterior lenticonus; fundus photography and optical coherence tomography showed bilateral giant macular holes which were larger than photographed at age 28. Electron microscopy of renal biopsy showed irregular thinned and thickened areas of the glomerular basement membrane with splitting of the lamina densa. Her son was then found to have hematuria (at age 3), and indirect immunofluorescence of the epidermal basement membrane showed negative staining for the collagen α5(IV) chain. However, the ophthalmological examinations of her son were unremarkable. A novel *COL4A5* mutation g. 4400_4400+1del, leading to an indel in exon 45 (r. 4198delins4198+2_ 4198+72), was detected in the patient and her son. This mutation produces a shift in the reading frame, resulting in a missense sequence of 13 codons followed by a premature stop codon. Her mother was not affected with the mutation.

**Conclusions:**

Our report extends the phenotypic and genotypic spectrum of X-linked Alport syndrome.

## Introduction

Alport Syndrome (AS) is a generalized inherited disease characterized by hematuria, progressive renal failure, sensorineural deafness and ocular abnormalities [1]. X-linked AS (XLAS, OMIM 301050), resulting from mutations in the type IV collagen alpha 5 (*COL4A5*) gene encoding the type IV collagen α5 chain, accounts for 85% of AS. Up to now, nearly 700 COL4A5 mutations have been reported (accessed May 6, 2012). About 45% of *COL4A5* mutations are missense mutations. The remaining mutations are splice site mutations, deletions, insertions, small indels, or complex rearrangements. However, these mutations are located throughout the gene without any identified mutational hot spot. The remainder of the AS patients have autosomal recessive or, rarely, an autosomal dominant inheritance (OMIM 203780, 104200), both of which result from mutations in the *COL4A3* or *COL4A4* gene [2-4]. Diagnosis of AS relies on clinical presentation, immunohistochemical analysis of collagen α (IV) chains in skin and/or renal biopsy specimen, ultrastructural changes of the glomerular basement membrane (GBM) and genetic molecular analysis.

Ocular abnormalities may involve the cornea, iris, lens, or retina in AS [2,4], among which anterior lenticonus and the dot-and-fleck retinopathy are characteristic of AS. Colville et al. [2] reported that the dot-and-fleck retinopathy occurred in 85% of affected adult males, and anterior lenticonus in approximately 25% of all XLAS patients. However, a lower incidence rate of retinopathy (about 20%) were observed in recent large-scale cohorts [3,5,6], one of which reported a strong relationship between *COL4A5* mutation categories and ocular changes in XLAS male patients [6]. Anterior lenticonus results from bulging of the lens through a thinned anterior capsule. Vertical dehiscences extending up to two thirds of the thickness of the anterior capsule were also observed under electron microscope [7-9]. Immunostaining pattern of α3 (IV) to α5 (IV) collagen chains in the anterior lens capsule ranged from decreased immunofluorescence to normal [9,10], reflecting a genetic heterogeneity [10]. Abnormal α3α4α5 type IV collagen network in the internal limiting membrane/nerve fiber layer (ILM/NFL) may be responsible for the pathogenesis of the dot-and-fleck retinopathy in AS patients [11].

Macular hole [12] and posterior lenticonus [13-16] are rare ocular manifestations in AS, and few reports described the underlying mutations. Macular hole accompanied with both anterior and posterior lenticonus has not previously been reported. In this study, we present a novel splice site mutation in the *COL4A5* gene identified in a Chinese female patient who presented with bilateral giant macular holes and bilateral anterior and posterior lenticonus.

## Methods

### Patient

**Figure 1:**
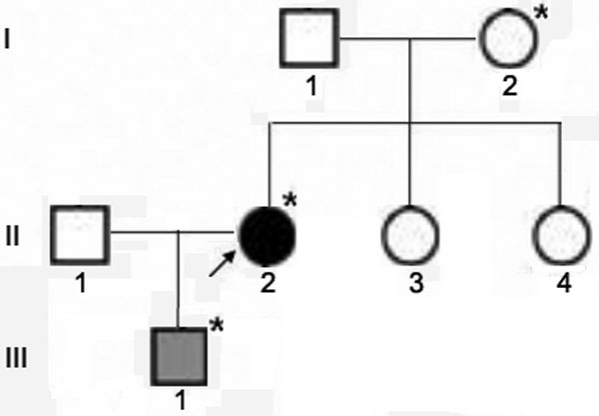
Pedigree of a Chinese family with Alport syndrome. The filled black circle indicates the individual with hematuria and proteinuria. The filled gray square indicates the individual with hematuria. The arrow indicates the proband. The asterisks indicate the individuals who had undergone molecular genetic analysis in the study.

**Figure 2:**
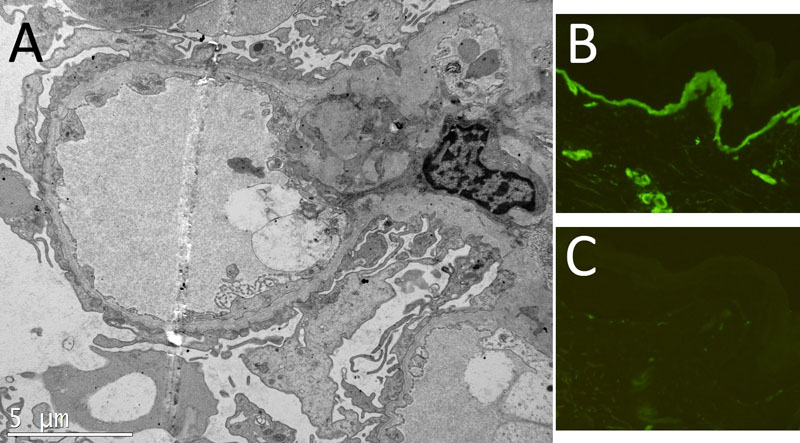
Electron micrograph of the renal biopsy and indirect immunofluorescence staining of the skin biopsy. Electron micrograph of the renal biopsy of the patient II-2 (**A**) showed irregular thinned and thickened areas of the GBM with splitting of the lamina densa (magnification, 8,000×). Indirect immunofluorescence staining of the skin biopsy using murine monoclonal antibodies against the NC domain of collagen α5 (IV) and α1 (IV; Wieslab, Lund, Sweden) showed that III-1 was positive for the α1 (IV) chain in a linear continuous pattern (**B**; positive control) and negative for the α5 (IV) chain (**C**), respectively (magnification, 200×).

A pedigree of the Chinese Alport kindred is shown in Figure 1. The female proband, II-2, initially had decreased visual acuity at age 14. At age 25, laboratory investigation showed microscopic hematuria. At age 33 in 2007, she was referred to our hospital. She had normal blood pressure, normal renal function (the serum level of creatinine was 74 μmol/l, and that of urea nitrogen was 4.10 mmol/l), microscopic hematuria with mild proteinuria (0.87 g/24 h), and sensorineural hearing loss on pure-tone audiometry; electron microscopy showed irregular thinned and thickened areas of the GBM with splitting of the lamina densa (Figure 2A). The proband’s only son, III-1, presented with hematuria at age 3 in 2009, and indirect immunofluorescence of the epidermal basement membrane showed positive for the collagen α1 (IV) chain and negative for the α5 (IV) chain (Figure 2B,C). The proband’s parents and siblings (I-1, I-2, II-3, and II-4) had no history of microscopic hematuria and proteinuria. After obtaining informed consent, a formal ophthalmological examination was performed in the proband and her family members. Gene analysis of *COL4A5* was performed in II-2, III-1 and I-2 with approval from the Ethical Committee of Peking University First Hospital. The proband denied any surgical intervention.

### Ocular examination

Proband II-2 underwent detailed ophthalmologic examinations including best corrected visual acuity (BCVA), near vision test, slit lamp examination of the anterior segment under maximally dilated pupil, direct and indirect ophthalmoscopy, fundus photography, optical coherence tomography (OCT; 3D OCT-1000; Topcon, Tokyo, Japan) at her first presentation to our hospital. Individual III-1 (at the age of 5), I-1, I-2, II-3, and II-4 also underwent all the above ophthalmologic evaluations. Digital slit lamp photography was used to document the bilateral anterior and posterior lenticonus found in proband II-2.

### *COL4A5* gene analysis

The entire coding region of *COL4A5* mRNA from cultured skin fibroblast of III-1 was analyzed by using reverse transcription-polymerase chain reaction (RT–PCR) and direct sequencing as described in our previous report [17]. The *COL4A5* mRNA transcripts in cultured skin fibroblasts of II-2 and III-1 were analyzed by RT–PCR which amplified a 284 bp fragment of the *COL4A5* cDNA encompassing exons 45, 46 and partial sequences of exons 44 and 47. A pair of primers (F, 5′-CTC CAG GTC CTC CTG GAT TA-3′; R, 5′-GGG ACC TTG CAA TCC ATC T-3′) was designed according to the published sequence (NM_033380). The PCR mixture (per 25 μl) contained cDNA template 1 μl, 2× Taq plus Master Mix (Tiangen Biotech Co., Ltd., Beijing, China) 12.5 μl, and 5 pmol/μl of each primer 1μl. The PCR parameters were optimized as follows: an initial denaturation at 94 °C for 7 min, 35 cycles of denaturation (94 °C for 30 s), annealing (58 °C for 30 s), elongation (72 °C for 45 s), and a final elongation for 7 min at 72 °C. The PCR products were checked by electrophoresis in a 2% agarose gel and by direct sequencing. Cultured skin fibroblast from two normal males were used as controls.

To further confirm the abnormality in exon 45 detected at the cDNA level, genomic DNAs of II-2, III-1, and I-2 were amplified using the published PCR primers and conditions for exon 45 [18] and were analyzed by direct sequencing.

## Results

### Findings on ocular examination

**Figure 3:**
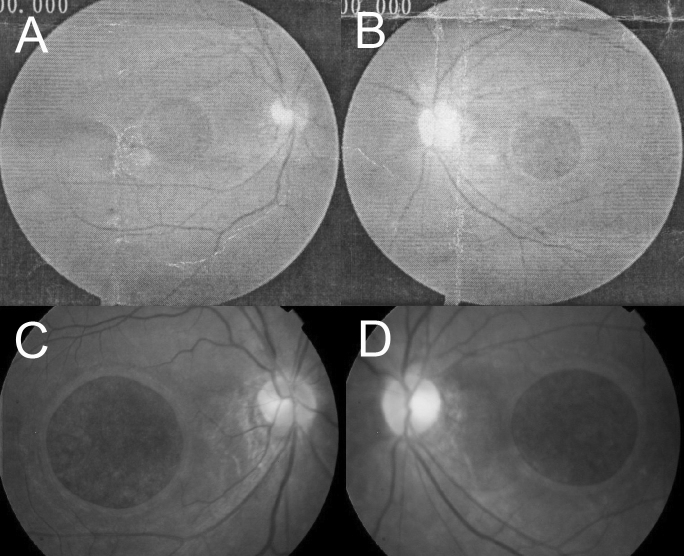
Slow progression of bilateral giant macular holes. The diameters of the macular holes were about 2 disc diameter at age 28 (**A**, **B**). At age 33, when first referred to our hospital, the diameters of the macular holes had increased to about 2.5 disc diameter (**C**, **D**).

**Figure 4:**
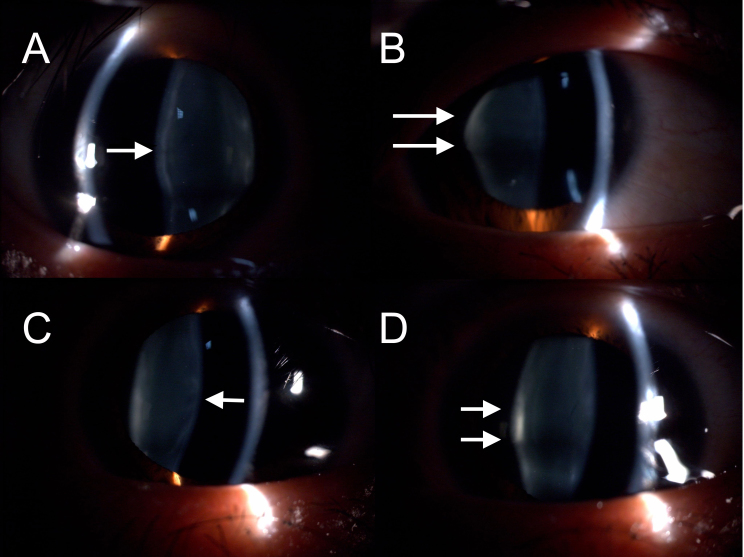
Bilateral anterior and posterior lenticonus. Digital slit lamp photography showed both anterior lenticonus (indicated with single arrows) and posterior lenticonus (indicated with double arrows) in both the right eye (**A**, **B**) and the left eye (**C**, **D**).

Proband II-2 had been diagnosed with progressive myopia in the local hospital since she first complained decreased visual acuity in both eyes at age 14. It was not until she was 28 years old that bilateral giant macular holes (about 2 disc diameter, DD) were documented in the local hospital (Figure 3A,B). When first presented to our hospital at age 33, her BCVA was 0.2 with −8.75 D sphere OU; her near vision was J2 OU; slit lamp examination of the anterior segment showed bilateral anterior and posterior lenticonus (Figure 4); fundus photography and OCT showed bilateral giant macular holes which were larger than photographed at age 28 (Figure 3C,D).

The ophthalmological examinations of III-1, I-1, I-2, II-3, and II-4 were unremarkable except that both of II-3 and II-4 were moderately myopic.

### Gene analysis

**Figure 5:**
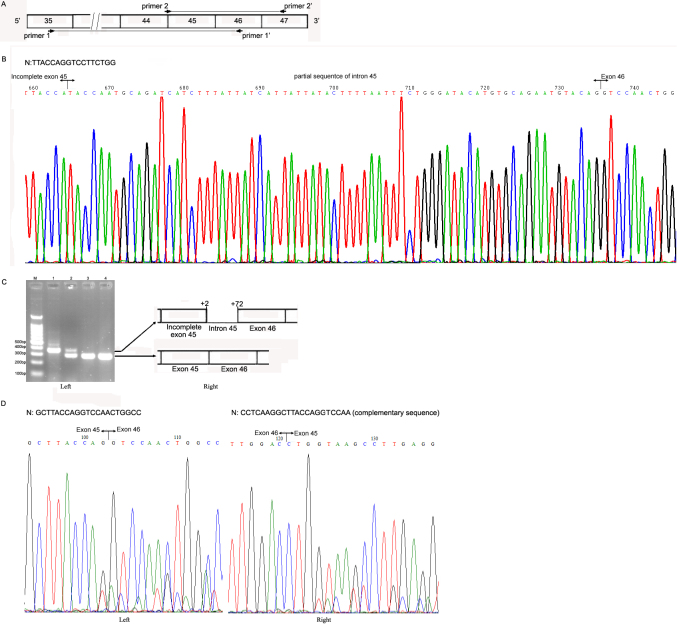
Sequencing and RT–PCR analysis of the cultured skin fibroblast mRNA. **A**: Strategy for amplification of the *COL4A5* cDNA by PCR. Exons are represented by open rectangles and are numbered. The localizations of the two amplified fragments are shown with the primer binding sites (horizontal arrows). **B**: Sequencing of the *COL4A5* cDNA amplified by a pair of primers 1/1’ in III-1. The junctions of exons 45, 46, and intron 45 are shown by the vertical lines, respectively. N: normal sequence. **C**: Left; RT–PCR amplification of a 284 bp fragment of *COL4A5* mRNA using a pair of primers 2/2’. M: DNA molecular mass marker; Lane 1 to 4: PCR products of *COL4A5* cDNA from III-1, II-2 and two normal male controls, respectively. As compared with the normal individuals, III-1 had a single larger sized RT–PCR product that contained a 71- base- pair indel identified in exon 45, whereas II-2 had two RT–PCR products: the normal sequence and the mutant sequence. **C**: Right; Schematic representation of the aberrant *COL4A5* cDNA resulting from the splice site mutation. Exons are represented by open rectangles and are numbered, and intron 45 is indicated by the horizontal line. **D**: Forward (Left) and reverse (Right) sequencing of the *COL4A5* cDNA amplified by a pair of primers 2/2’ in II-2. The junctions of exons 45 and 46 are shown by the vertical lines, respectively. N: normal sequence.

As shown in Figure 5B, in III-1, direct sequencing of *COL4A5* cDNA, amplified by a pair of primers 1/1’ (Figure 5A), revealed an indel in exon 45 (r. 4198delins4198+2_ 4198+72). Agarose gel electrophoresis showed that he had a larger sized RT–PCR product, suggestive of an insertion; as for II-2, a heterozygous pattern of PCR products was detected (Figure 5C). It is worth noting that the mRNA expression level of the normal *COL4A5* allele was higher than the mutant. Subsequent bidirectional sequencing of II-2’s RT–PCR product confirmed that she was heterozygous with one normal allele and one mutated allele (Figure 5D).

**Figure 6:**
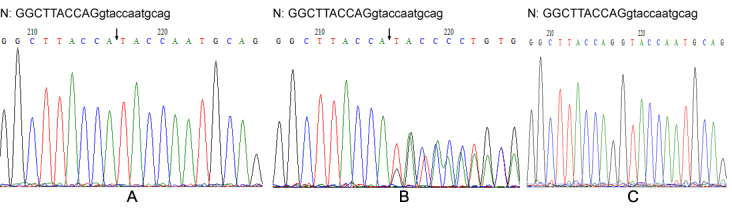
Genomic DNA sequencing of exon 45 in the *COL4A5* gene. Sequencing of PCR-amplified products of exon 45 from III-1, II-2, and I-2 are shown in **A**, **B**, and **C**, respectively. N: normal sequence. Exon and intron sequences are depicted by capital and lower case letters, respectively. The breakpoint is indicated by the arrows.

To further characterize the mutation, *COL4A5* exon 45 with sequences of flanking introns were ampliﬁed by PCR from genomic DNAs of II-2 and III-1; subsequent sequence analysis demonstrated a hemizygous 2-base-pair deletion involving both the 3′ end of exon 45 and the 5′ end of intron 45 in III-1 (Figure 6A); II-2 was found to be heterozygous for the same mutation (Figure 6B). This data confirmed the result of cDNA analysis. Namely, mutation g. 4400_4400+1del (numbered according to the reference sequence deﬁned by Zhou et al. [19]) resulted in a single base deletion at position 4198 and affected the 5′ splice site, which caused an insertion of 71-bp of intron 45 between nucleotides 4197 and 4199 of the *COL4A5* mRNA (NM_ 000495). This mutation produces a shift in the reading frame, resulting in a missense sequence of 13 codons followed by a premature stop codon. According to the Human Gene Mutation database (accessed May 6, 2012), the mutation was novel. I-2’s DNA was negative for mutation g. 4400_4400+1del screen (Figure 6C).

## Discussion

According to the strict diagnostic criteria of AS proposed by Flinter et al. [20], the proband described here could be clinically diagnosed with XLAS. An identified splice site mutation in *COL4A5* was sufficient to explain the development of renal disease, deafness and ocular abnormalities.

Both macular hole and posterior lenticonus are rare ocular manifestations in AS [12-16]. Bekheirnia et al. [6] reported that macular hole occurred in 1 out of 681 US male patients with XLAS and characterized the exact pathogenic mutation. Shah et al. [12] reviewed 9 patients with large macular holes and identified AS in 6 of these cases, wherease the defect in *COL4A5* gene was unknown. Posterior lenticonus usually coexist with anterior lenticonus [13-16], and no genotypic data on AS patients with posterior lenticonus is currently available. II-2 presented with not only anterior and posterior lenticonus, but also macular holes. To our knowledge, this is the first report of such rare ocular phenotype in XLAS.

II-2 had no visual complants until 14 years old, suggesting a late adolescence or early adulthood onstet of macular holes, which was in accordance with previous reports [12,21-23]. Currently, few reports have observed the dynamic evolution of macular holes, except that Shah et al. [12] documented the evolution of multiple small macular holes into a large macular hole in a male AS patient. In II-2, enlargement of the macular holes were observed from age 28 to 33, demonstrating the slowly progressive nature even in giant macular holes (2 DD in diameter). The pathogenesis of macular holes in AS patients is not clear. Dysfunction of the Bruch membrane [24] and the internal limiting membrane [12] due to abnormal type IV collagen were prosposed to be involved. Although anterior lenticonus has long been recognized as a characteristic ocular manifestation of AS, posterior lenticonus has rarely been reported to be associated with AS [16]. The preferential involvement of the anterior lens capsule in AS remains to be investigated. Detailed ocular status should be monitored regularly in II-2 and III-1, which might provide further information on pathogenesis, nature history, prognosis and potential intrafamilial heterogeneity of this specific ocular phenotype.

A strong relationship has been observed between *COL4A5* mutation categories and ocular changes in European and US male patients with XLAS. In the study by Jais et al. [3], the frequency of lenticonus was significantly higher in male patients with large *COL4A5* deletion or small mutation resulting in premature stop codon. In the study by Bekheirnia et al. [6], male patients with *COL4A5* deletions or splice or truncating mutations had two- to fourfold greater odds of developing ocular abnormalities than those with missense mutations. In II-2, the identified splice site mutation in *COL4A5* was predicted to produce a truncated α5 (IV) chain lacking the normal NC domain and part of the collagenous domain, which would interfere with the formation of the triple helical structure of type IV collagen molecule and the assembly of the supramolecular type IV collagen structure. As in the eye, the α5 (IV) chain is normally present in the lens capsule, Descemet, Bruch and internal limiting membranes [25], it is reasonable to infer that dysfunction of the lens capsule, Bruch and internal limiting membranes resulted from deposit of abnormal type IV collagen was responsible for this severe ocular phenotype in II-2. III-1, however, presented with no ocular abnormalities, one possible explanation is that he was too young.

This rare ocular phenotype may also be due, in part, to the X-inactivation pattern, a process ensuring only a single X is active in both the XX female and the XY male. We previously reported that the quantity of the mRNA expression level of mutant *COL4A5* gene was correlated with the phenotypic severity of XLAS females [26]. A severe phenotype of two XLAS female patients was attributable to a severe skewed pattern of X-inactivation in favor of expression of the mutant *COL4A5* [27,28]. Rheault et al. [29] reported that in murine XLAS, favorable X-inactivation improved survival of female carriers. Unfortunately, the difficulty to obtain the eye tissues has impeded further study of the X-inactivation pattern of II-2.

In conclusion, this report described a rare ocular phenotype in a female XLAS patient and characterized a novel splice site mutation in the *COL4A5* gene, which provides new phenotypic and genotypic information on the disease. To further understand the molecular mechanisms of XLAS, a multidisciplinary strategy is recommended.
